# Duplication and Diversification of the Spermidine/Spermine N^1^-acetyltransferase 1 Genes in Zebrafish

**DOI:** 10.1371/journal.pone.0054017

**Published:** 2013-01-11

**Authors:** Yi-Chin Lien, Ting-Yu Ou, Yu-Tzu Lin, Po-Chih Kuo, Han-Jia Lin

**Affiliations:** 1 Institute of Bioscience and Biotechnology, National Taiwan Ocean University, Keelung, Taiwan; 2 Center of Excellence for Marine Bioenvironment and Biotechnology, National Taiwan Ocean University, Keelung, Taiwan; Institute of Molecular and Cell Biology, Singapore

## Abstract

Spermidine/spermine N^1^-acetyltransferase 1 (Ssat1) is a key enzyme in the polyamine interconversion pathway, which maintains polyamine homeostasis. In addition, mammalian Ssat1 is also involved in many physiological and pathological events such as hypoxia, cell migration, and carcinogenesis. Using cross-genomic bioinformatic analysis in 10 deuterostomes, we found that *ssat1* only exists in vertebrates. Comparing with mammalian, zebrafish, an evolutionarily distant vertebrate, contains 3 homologous *ssat1* genes, named *ssat1a, ssat1b*, and *ssat1c*. All zebrafish homologues could be transcribed and produce active enzymes. Despite the long history since their evolutionary diversification, some features of human SSAT1 are conserved and subfunctionalized in the zebrafish family of Ssat1 proteins. The polyamine-dependent protein synthesis was only found in Ssat1b and Ssat1c, not in Ssat1a. Further study indicated that both 5′ and 3′ sequences of *ssat1b* mediate such kind of translational regulation inside the open reading frame (ORF). The polyamine-dependent protein stabilization was only observed in Ssat1b. The last 70 residues of Ssat1b were crucial for its rapid degradation and polyamine-induced stabilization. It is worth noting that only Ssat1b and Ssat1c, but not the polyamine-insensitive Ssat1a, were able to interact with integrin α9 and Hif-1α. Thus, Ssat1b and Ssat1c might not only be a polyamine metabolic enzyme but also simultaneously respond to polyamine levels and engage in cross-talk with other signaling pathways. Our data revealed some correlations between the sequences and functions of the zebrafish family of Ssat1 proteins, which may provide valuable information for studies of their translational regulatory mechanism, protein stability, and physiological functions.

## Introduction

Polyamines, found in the cells of most species, play vital roles in cell proliferation and many physiological functions [1]. Thus, cellular polyamine homeostasis is strictly maintained by regulation of its anabolic and catabolic pathways [2]. In mammals, the interconversion pathway enhances control of cellular polyamine. Spermidine/spermine N^1^-acetyltransferase 1 (Ssat1) is the key enzyme in the rate-determining reactions of this pathway, by which spermine or spermidine accepts the acetyl group from acetyl-CoA to produce N^1^-acetylspermine or N^1^-acetylspermidine [3]. Ssat1 effectively reverses the biosynthetic reactions and alters cellular polyamine equivalence because the acetylated derivatives are readily excreted and are good substrates for acetylpolyamine oxidase (Apao), a peroxisomal enzyme that readily converts these molecules to smaller polyamines [4], [5].

Accumulating evidence has indicated that mammalian Ssat1 is also involved various physiological and pathological events, including liver regeneration [6], ischemia-reperfusion injury [7], [8], [9], pancreatitis [10], [11], lipid metabolism [12], [13], carcinogenesis [14], cell migration [15], and hypoxia signaling [16], through its ability to modulate polyamine content or by directly interacting with other protein effecters, such as hypoxia inducible factor 1-α (Hif-1α) and integrin α9. Moreover, the regulation of Ssat1 is as versatile as its functions, occurring at multiple levels including transcription [17], [18], mRNA processing [19], [20], [21], translation [22], and protein stabilization [23], [24], [25]. Curiously, we know rather little about the properties of Ssat1 from non-mammalian species. Here, we found that *ssat1* is only present in the vertebrate lineage. Comparing with mammalians, zebrafish, an evolutionarily distant vertebrate, contains not one but three *ssat1* genes. To understand whether these *ssat1* genes have evolved distinct structural and functional properties, their spatial and temporal expression, translational regulation inside the ORF, protein stability, enzyme kinetics, and protein-protein interactions were extensively investigated. Our data suggest that these zebrafish *ssat1* homologues might be paralogous genes which underwent subfunctionalization in their regulatory mechanisms and physiological functions.

## Materials and Methods

### General Materials

All chemicals, including 5,5′-dithio-bis-(2-nitrobenzoic acid), coenzyme A, acetyl-CoA, cycloheximide, DMSO, isopropyl β-D-1-thiogalactopyranoside, and polyamines were purchased from Sigma-Aldrich Chemical Co. and were of the highest purity available. N^1^,N^11^-diethylnorspermine (DENSPM) was from Tocris Bioscience. Enzymes used in molecular cloning were obtained from New England Biolabs. The pGEX-2T expression vector was from GE Healthcare Bioscience; the pcDNA3.1/myc-His vector was from Invitrogen; the pET28 expression vector and the *Escherichia coli* BL21 (DE3) host cells were from Novagen.

### Database Searches and Phylogenetic Analysis

**Figure 1:**
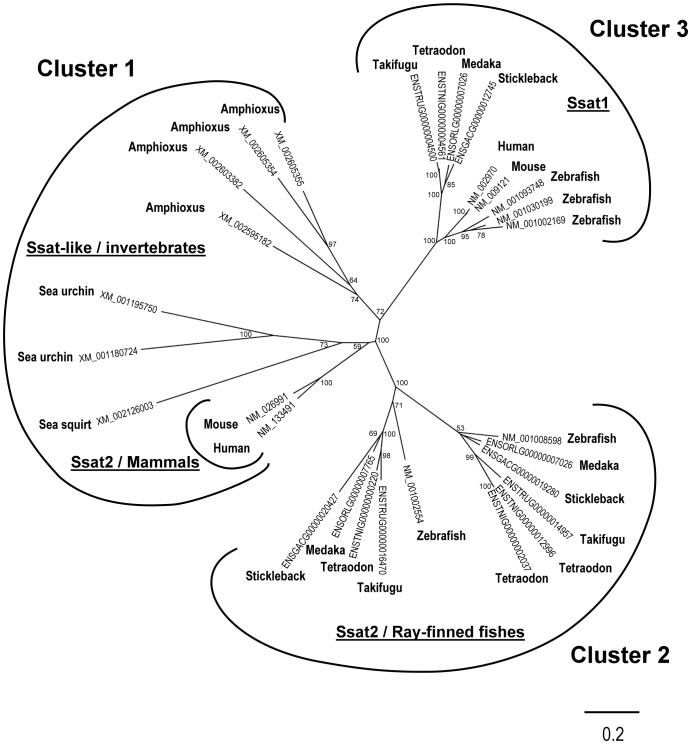
Phylogenetic analysis of *ssat-like* genes. The accession number of each *ssat-like* gene from the deuterostomia is denoted and the bars represent their evolutionary distance. The scale bar is 0.2 expected changes per amino acid site. The reliability of the tree was measured by bootstrap analysis. Bootstrap values of 1,000 replicates larger than 50 were labeled on branches.

In order to investigate the presence of SSAT1-related genes in invertebrate and vertebrate deuterostomes, human SSAT1 (NM_002970) and SSAT2 (NM_133491) were used as templates for TBlastN searches at the National Center for Biotechnology Information (NCBI) website (http://www.ncbi.nlm.nih.gov/mapview/) for sea urchin (*Strongylocentrotus purpuratus*), sea squirt (*Ciona intestinalis*), zebrafish (*Danio rerio*), and mouse (*Mus musculus*) genomes, or in Ensembl (http://www.ensembl.org/index.html) for medaka (*Oryzias latipes*), stickleback (*Gasterosteus aculeatus*), Takifugu (*Takifugu rubripes*), and Tetraodon (*Tetraodon nigroviridis*) genomes. The database hits were verified by BlastP queries of the NCBI nonredundant (nr) protein database and sequence alignments, before a putative homolog (accession numbers provided in Fig. 1) was considered.

The amino acid sequences of *ssat1*-related genes were aligned by ClustalW [26] and edited manually using the BioEdit software [27] to prepare for the phylogenetic analysis. The alignment is available upon request. Phylogenetic trees were constructed by the neighbor joining (NJ) method using the PHYLIP package [28] with the substitution model and represented with the FigTree software (http://tree.bio.ed.ac.uk/software/figtree/). Tree topology was evaluated by bootstrap resampling with 1000 replicates.

### Reverse Transcription (RT)-PCR and Molecular Cloning

Total RNA was isolated from human *HEK293T* cells (CRL-11268), adult zebrafish, and embryos with TRIzol reagent (Invitrogen). After DNase treatment, total RNA (500 ng) was reverse-transcribed to single-strand cDNA using SuperScript II reverse transcriptase (Clontech) according to the manufacturer’s instructions. Transcription of each gene was detected by RT-PCR with specific primers (see Table S1 in the supplemental material). The PCR reaction was initiated with DreamTaq (Fermentas) according to the manufacturer’s protocol. The reactions were separated by 1% agarose gel electrophoresis and visualized by staining with ethidium bromide.

To prepare expression plasmids for zebrafish *ZF4* (ATCC CRL-2050) or *HEK293T* cells, the full-length ORF of human *SSAT1*, zebrafish *ssat1a*, *ssat1b*, and *ssat1c*, or DNA fragments encoding the cytosolic domain of zebrafish *integrin 9α* and the PAS-B domain of *hif-1α* were amplified by RT-PCR with specific primer sets (Table S1) and *Pfu* DNA polymerase (Stratagene). These amplified inserts were ligated into the *Eco*RI/*Xho*I sites of pcDNA3.1/myc-His. The recombinant plasmids were transformed into *E. coli* for preservation and amplification.

**Figure 2:**
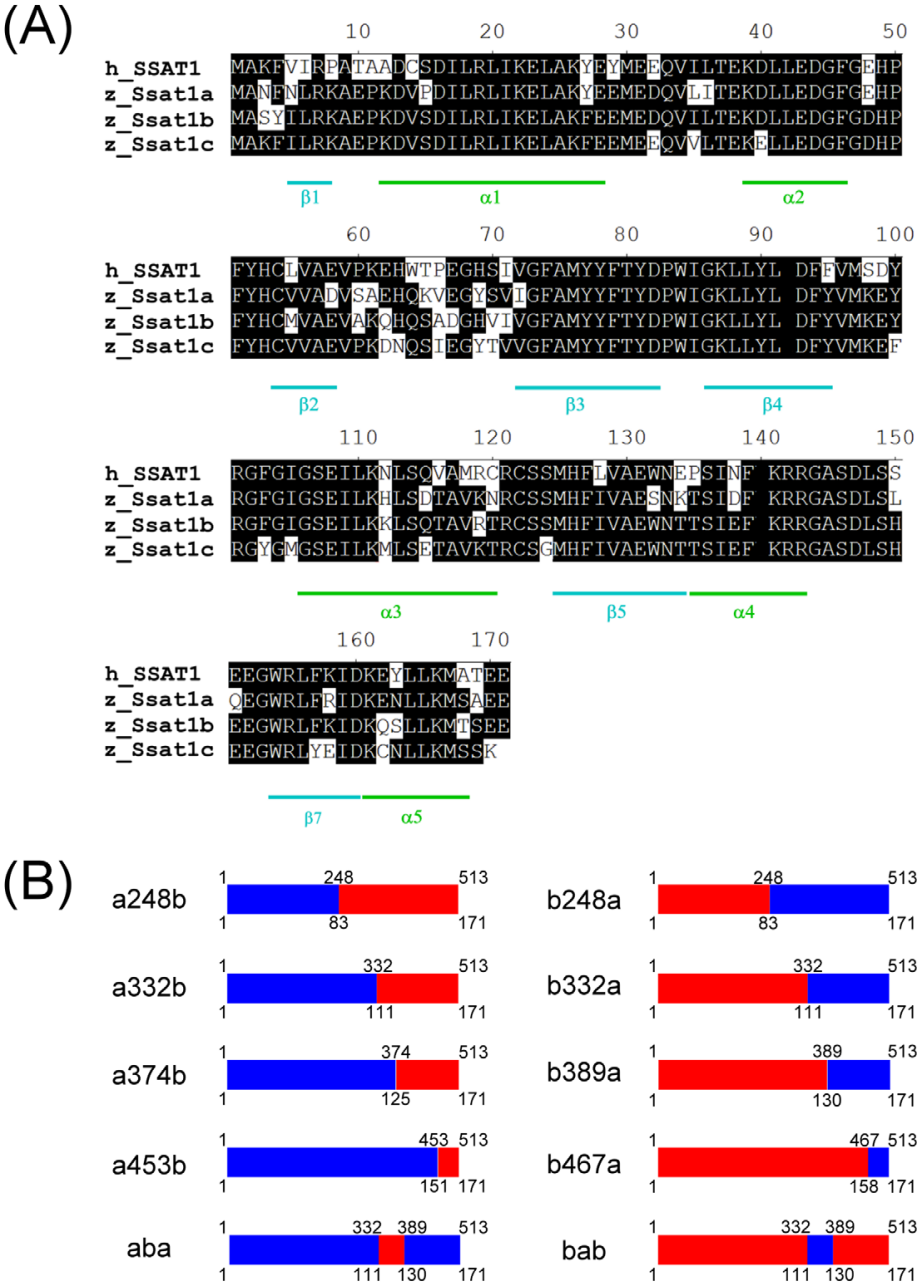
Primary structures of zebrafish family of Ssat1 proteins and the constructs of chimeric proteins used in this study. (A) The amino acid sequences of human SSAT1 and zebrafish family of Ssat1 proteins were aligned by MegAlign (Lasergene) with the ClustalW method. The conserved residues are shaded black. The secondary structures are denoted according to the structure of human SSAT1 [33]. (B) In each chimeric construct, fragments from Ssat1a are labeled in blue and fragments from Ssat1b are in red. Nucleotide positions and the corresponding amino acid residues are labeled on the top and the bottom of each construct, respectively.

The chimeric *ssat1a* and *ssat1b* genes were prepared by using the megaprimer PCR technique [29]. We designed 4 chimeric primers, which could pair to the same region of *ssat1a* and *ssat1b* at nucleotides 235–248, 319–332, 374–389 and 452–467 (Table S1). To make *ssat1a248b*, for example, the chimeric primer (primer 11 in Table S1) and *ssat1a* forward primer (primer 3 in Table S1) were used to amplify the target DNA fragment (nucleotides 1–248 of *ssat1a*). Then, the 2 strands of newly synthesized PCR fragments were used as megaprimers with *Pfu* DNA polymerase to synthesize the whole plasmid (pcDNA3.1/myc-His-*ssat1b*) and incorporate the chimeric target DNA fragments. The original plasmid DNA was digested by *Dpn*I, and the *Dpn*I-resistant chimeric DNA was recovered by transformation into competent bacteria. The chimeric gene was named *ssat1a248b*, as it contains nucleotides 1–248 of *ssat1a* and the remainder of *ssat1b*. Chimeric genes *ssat1a332b*, *ssat1a374b*, *ssat1a453b*, *ssat1b248a*, *ssat1b332a*, *ssat1b389a*, and *ssat1b467a* were also prepared in this manner (Fig. 2B). The chimeric gene *ssat1aba* was prepared by using nucleotides 389–513 of *ssat1a* as the megaprimer and a plasmid containing *ssat1a332b* as template. Therefore, *ssat1aba* and *ssat1bab* contain most of the *ssat1a* and *ssat1b* sequences, but their 332–389 nucleotide regions are from *ssat1b* and *ssat1a*.

### Preparation of Recombinant Protein and Enzyme Activity Assay

The coding sequence of human *SSAT1*, zebrafish *ssat1* homologues, and the *integrin α9* cytosolic, and *hif-1α* PAS-B domains were amplified (Table S1) from the cDNA of *HEK293T* cells or zebrafish embryos. PCR products were inserted into the *Eco*RI and *Xho*I sites of pET28a or pGEX2T and transformed into *E. coli* BL21 to express recombinant proteins with N-terminal His_6_-tag or GST fusions. Following cell lysis, the His-tagged and GST-fused enzymes were purified by affinity chromatography on a HisTrap FF column (GE Healthcare) and a GSTrap FF column (GE Healthcare), respectively. The SSAT1 activity assay was described in our previous work [30].

### Cell Culture, Gene Transfection, and Cellular Protein Extraction

Zebrafish *ZF4* cells were cultured in 45% DMEM (Gibco), 45% Ham’s F12 medium (Gibco), and 10% FBS (Gibco) at 28°C with humidified air/CO_2_ (19∶1 v/v). Human *HEK293T* cells (CRL-11268) were cultured in DMEM with 10% FBS at 37°C with humidified air/CO_2_ (19∶1 v/v). The *HEK293T* cells were transfected by using standard calcium phosphate precipitation and transfection of *ZF4* cells was performed as described previously [30]. To prepare cellular proteins for analysis, cells were collected by centrifugation at 300×*g*, washed twice with PBS, and extracted with M-PER reagent (Thermo Scientific) according to the manufacturer’s instructions. The crude extract was dialyzed against 50 mM Tris-HCl (pH 7.5) with 1× Protease inhibitor (complete protease inhibitor cocktail, Roche) with an Amicon ultra centrifugal filter device (Millipore).

### Detection of Protein Expression and Degradation by Western Blotting

*HEK293T* cells were seeded at a density of 3 × 10^5^/well (6-well plate) and transfected with 2 µg pcDNA3.1/myc-His plasmids containing the ORFs of human *SSAT1*, zebrafish *ssat1a*, *ssat1b*, *ssat1c*, or chimeric genes. After 24 h culture, cells were treated with 10 µM DENSPM, 5 µM MG132 or vehicle (DMSO), and incubated for another 24 h before harvest and detection of translated proteins. To assess protein stability, cells were transfected with 2 µg plasmid encoding Ssat1a, Ssat1aba, Ssat1a453b, or Ssat1b332a, or with 4 µg plasmid encoding Ssat1b, Ssat1c, Ssat1bab, Ssat1b332a, or Ssat1b467a. After 12 h culture, cells were treated with 200 mM cycloheximide or left untreated. Before harvest, cells were treated with 2 mM spermidine for 2–6 h in the presence of 10 µM MG132 or vehicle (DMSO).

Cell lysates were resolved by 12% SDS–PAGE and transferred to a PVDF membrane. Proteins were immunodetected with anti-myc primary (1∶2000, Cell Signaling) and anti-mouse IgG secondary antibodies (1∶5000, Promega). Signals were detected with ECL Plus chemiluminescence reagent (GE Healthcare) and an imaging system (UVP Biospectrum).

### GST Pull-down Assay

*HEK293T* cells (3×10^6^ cells/10-cm plate) were transiently transfected with expression vectors encoding myc-tagged Ssat1a, Ssat1b, Ssat1c, or the PAS-B domain of Hif-1α. Cells transfected with Ssat1a, Ssat1b and Ssat1c expression vectors were cultured in the medium with 10 µM DENSPM. After 48 h culture, cell lysates (100 µg) were harvested and mixed with 10 µg GST or GST fusion proteins in 500 µl PBS buffer at 4°C for 2 h, followed by addition of 20 µl of glutathione-Sepharose 4B beads (GE Healthcare). After mixing for 30 min, the beads were washed with PBS. The proteins were eluted in Laemmli sample buffer and analyzed by SDS-PAGE and western blotting.

## Results

### Identification of *ssat1*-like Genes in Deuterostomes

Several *ssat*-like genes were found across the deuterostomia, including sea urchin, sea squirt, amphioxus, mouse, human, and 5 kinds of ray-finned fish. Phylogenetic analysis divided these genes into 3 clusters (Fig. 1). Cluster 1 was composed of human *SSAT2*, mouse *Ssat2*, and invertebrate *ssat*-like genes. Genes in cluster 2 were *ssat2* orthologues from ray-finned fish. At least 2 *ssat2* homologous genes were found in all ray-finned fish species analyzed in this study. These *ssat2* homologues were further divided into 2 sub-groups that suggested an early duplication event at *ssat2* in the common ancestor of ray-finned fishes. Cluster 3 included human *SSAT1* and its cognate genes from vertebrates. Compared with the first 2 clusters, the *ssat1* orthologues were more closely conserved. No *ssat1* orthologue was found in invertebrates and only 1 *ssat1* was found in most vertebrates except that there were 3 *ssat1* homologues in zebrafish. The encoded amino acid sequences (Fig. 2A) and cDNA sequences (Fig. S2) of zebrafish *ssat1* homologous genes were highly similar to each other, and they were clustered together in the phylogenitic analysis (Fig. 1).

Human *SSAT1* is located on the X chromosome between the genes for peroxiredoxin 4 (*PRDX4*), acryl-CoA thioesterase 9 (*ACOT9*), and apolipoprotein O (*APOO*) (Fig. S1). The *Ssat1* genes of evolutionarily distant vertebrates including medaka, stickleback, takifugu, and tetraodon are located between *acot9* and *apoo* (data not shown). One of the zebrafish *ssat1*-like genes (NM_001093748) is also located between *acot9* and *apoo* on chromosome 24; we therefore named it *ssat1a*. The other zebrafish genes (NM_001030199 and NM_001002169) are closely clustered together and located next to *prdx4* on chromosome 5. We named them *ssat1b* and *ssat1c*, respectively. The *ssat*-like genes of invertebrates and *ssat2* homologous genes of vertebrates are not grouped like *ssat1* (Fig. 1) and their genomic localization also differ (data not shown).

### The Expression Pattern of Zebrafish *ssat1* Homologous Genes

**Figure 3:**
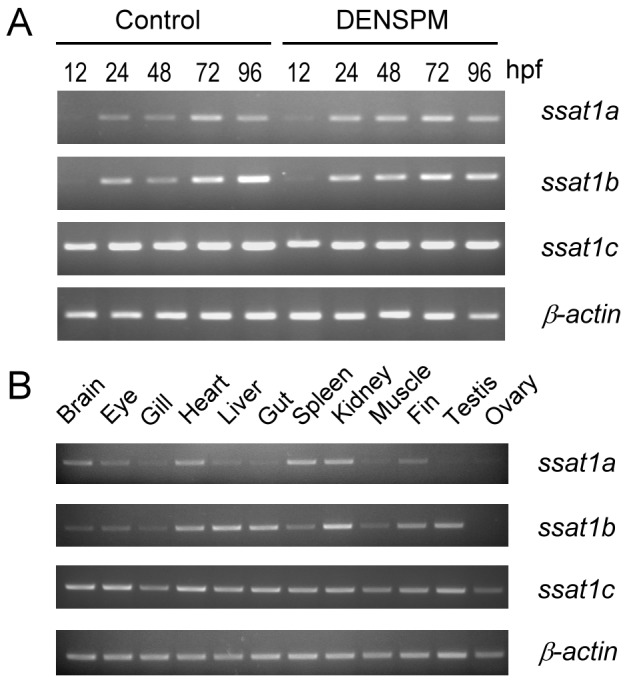
Temporal and spatial expression patterns of zebrafish *ssat1* homologous genes. (A) After fertilization, zebrafish embryos were incubated with or without (control) 10 µM DENSPM. Embryos were collected at different developmental stages (shown on the top, hpf, hour post fertilization) and expression of *ssat1a*, *ssat1b*, *ssat11c*, and *β-actin* were analyzed by RT-PCR. (B) The expression patterns of zebrafish *ssat1* homologous genes were also analyzed in the major organs (shown on the top) of adult zebrafish.

The expression patterns of zebrafish *ssat1* genes were analyzed by RT-PCR. During normal embryogenesis, *ssat1c* mRNA was the most abundant in every stage and was stably expressed from 12 to 96 hours post fertilization (hpf). The mRNA of *ssat1a* and *ssat1b* were not detected until 24 hpf (Fig. 3A, control). A previous study indicated that treatment of human cells with DENSPM, a spermine analog, enhances *SSAT1* expression up to 20 fold [31]. Another group of zebrafish embryos were developed with 10 µM DENSPM added immediately after fertilization. All embryos survived and displayed no obvious abnormalities through 96 hpf. Neither the expression nor mRNA abundance of these *ssat1* genes was changed (Fig. 3A, DENSPM).

The expression profiles of zebrafish *ssat1* genes in the major organs of adult fish were also studied. *ssat1a* mRNA was mainly expressed in the heart, spleen and kidney, and was also detectable in the brain, eye, liver, and fin. *ssat1b* mRNA was detectable in the heart, liver, gut, and kidney, and weakly in the brain, eye, gill, spleen, muscle, fin and testis. *ssat1c* mRNA was detectable in every organ we tested and was the most abundant among these three homologues (Fig. 3B).

### The Translational Regulation Inside the ORF of Zebrafish *ssat1* Homologues

**Figure 4:**
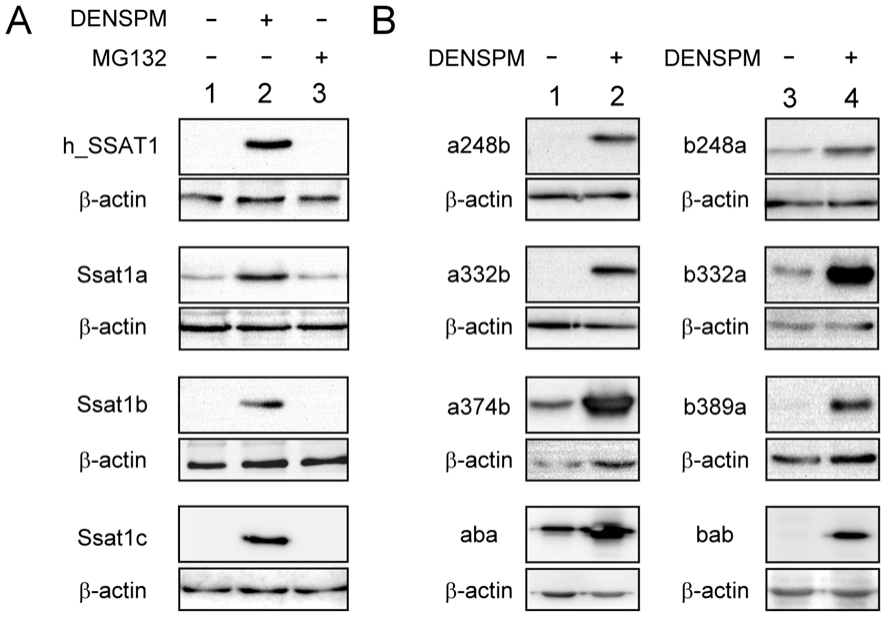
Translational regulation inside the ORF of zebrafish family of Ssat1 proteins. (A) *HEK 293T* cells were transiently transfected with the plasmid encoding myc-tagged full-length human SSAT1, zebrafish Ssat1a, Ssat1b, or Ssat1c. After incubation for 12 h, transfected cells were treated with 10 µM DENSPM, 20 µM MG132, or vehicle for 24 h. (B) *HEK 293T* cells were transiently transfected with the plasmid encoding myc-tagged zebrafish Ssat1 chimeric enzymes (detail in Materials and Methods). After incubation for 12 h, transfected cells were treated with vehicle (lane 1 and 3) or 10 µM DENSPM (lane 2 and 4) for 24 h. Cell lysates (5 µg total protein in each lane) were prepared and the protein content of Ssat1 and β-actin in each sample was detected by western blotting with anti-myc and anti-β-actin antibody.

It has been reported that the translation of human SSAT1 is strictly controlled by polyamine and the *SSAT1* ORF region is responsible for such regulation [22], [32]. To test this mechanism, the ORF of each gene was ligated into pcDNA3.1/myc-His, which allows the mRNA of each gene to be stably and abundantly expressed. Human SSAT1 protein was extensively expressed in transfected *HEK293T* cells incubated with DENSPM but not in cells cultured in normal medium or medium with MG132 (Fig. 4A). Addition of MG132, a proteasome inhibitor, may increase SSAT1 protein stability but did not increase protein abundance in the absence of DENSPM. This is similar to previous studies, which suggested that the activity of SSAT1 is mainly regulated by polyamine and polyamine analogs in the translation level [22], [23].

We investigated the translational regulation inside the ORF of zebrafish *ssat1* under the same experimental conditions. It is interesting to note that background expression of Ssat1a was detectable in cells cultured without DENSPM and its expression increased by approximately 3-fold in the presence of DENSPM. On the other hand, the translational regulation inside the ORF of *ssat1b* and *ssat1c* were as stringent as that of human *SSAT1* (Fig. 4A). We performed the same experiment in zebrafish *ZF4* cells that also obtained the same result (Fig. S3). Thus, the *ssat1* translational regulation machinery appears to be conserved in human and fish cells.

In order to identify the key region for translational regulation inside the ORF, a series of chimeric genes (*ssat1a248b*, *ssat1a332b*, *ssat1a374b* and *ssat1a453b*), which contained the 5′ region of *ssat1a* ORF and the 3′ region of *ssat1b*, were prepared and tested (Fig. 2B). The results indicated that the last 181 nucleotides of *ssat1b* are important for translational inhibition, since chimeric genes containing more than 181 nucleotides from the 3′ region of *ssat1b* retained the translational regulation pattern of *ssat1b*, such as *ssat1a248b* and *ssat1a332b* (Fig. 4B, lanes 1 and 2). The results of another series of chimeric mRNA with the 5′ region of *ssat1b* and the 3′ region of *ssat1a* (*ssat1b248a*, *ssat1b332a*, *ssat1b389a* and *ssat1b467a* shown in Fig. 2B) showed that the first 389 nucleotides of *ssat1b* are also important for regulation (Fig. 4B, lanes 3 and 4). Because the 332∼389 nucleotide region of *ssat1b* is present in both *ssat1a332b* and *ssat1b389a*, the importance of this region was further investigated.

The chimera *ssat1aba*, which contains the 332∼389 region of *ssat1b* and the remainder of *ssat1a,* still retained the *ssat1a* regulatory pattern that suggests the 332∼389 region of *ssat1b* alone is not sufficient to inhibit the background protein translation (Figure 4B). Further, *ssat1bab*, which contains the 332∼374 nucleotide region of *ssat1a* and the remainder of *ssat1b*, behaved like *ssat1b*. These results, which are summarized in table 1, indicate that both the 5′ and 3′ regions of *ssat1b* are important for translational regulation inside the ORF.

**Table 1:** Translational regulation inside the ORF and protein stability regulation of each gene in response to polyamine.

	hSSAT1	zSsat1a	zSsat1b	zSsat1c	a248b	a332b	a374b	a453b	aba	b248a	b332a	b389a	b467a	bab
Translational regulation inside the ORF	+	Δ*	+	+	+	+	Δ	Δ	Δ	Δ	Δ	+	+	+
Protein stabilityregulation	+**	−	+	−	+	+	−	−	−	−	−	−	−	−

* Triangle marks indicate genes with similar translational regulation pattern as zSsat1a in Fig. 4A. They maintained basal level protein translation in the DENSPM free culture condition.

** Data from Coleman *et al.* 2001 [23].

### The Stability of Zebrafish *Ssat1* Proteins

**Figure 5:**
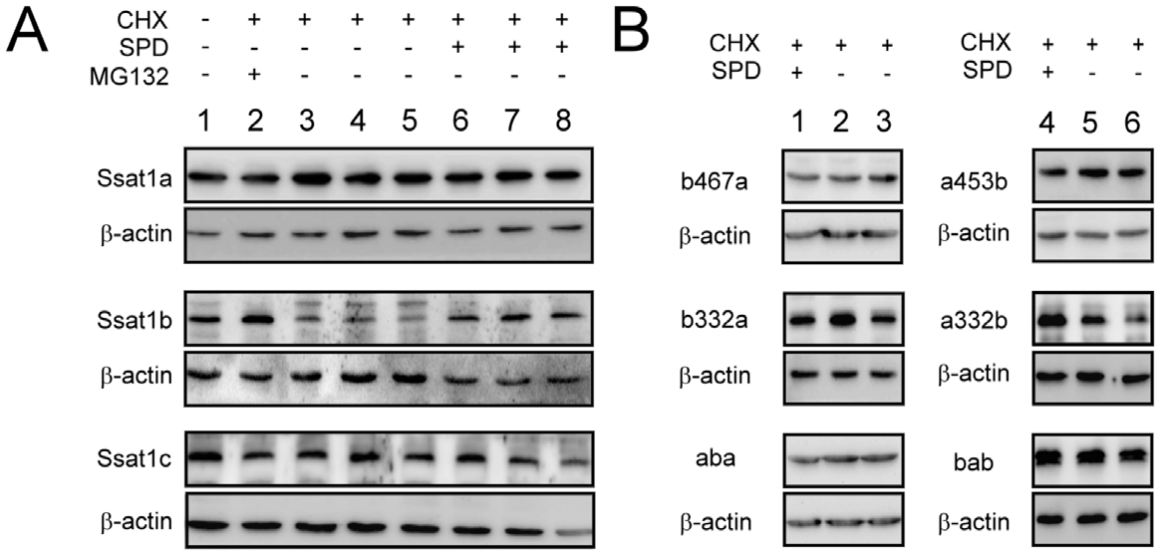
Protein stability of Ssat1b was regulated by polyamine. (A) *HEK 293T* cells were transiently transfected with the plasmid encoding full-length zebrafish Ssat1a, Ssat1b, or Ssat1c. After 24 h, cells were treated with 200 mM cycloheximide (CHX) or left untreated for 30 min (lane 1). Then cells were treated with 10 µM MG132 (lane 2), vehicle (lanes 3–5), or 2 mM spermidine (lanes 6–8) for 1 h (lanes 3 and 6), 2 h (lanes 4 and 7), or 4 h (lanes 2, 5 and 8). (B) *HEK 293T* cells were transiently transfected with the plasmid encoding myc-tagged zebrafish Ssat1 chimeric enzymes (details in Materials and Methods). After incubation for 24 h, transfected cells were treated with 200 mM cycloheximide (CHX) for 30 min (lanes 1 and 4) and then with 2 mM spermidine for 1 h (lanes 2 and 5) or 2 h (lanes 3 and 6). Cell lysates (50 µg total protein in the Ssat1b, Ssat1c, b467a, a332b, and bab samples; 5 µg of total protein in the remaining samples) were prepared and the Ssat1 and β-actin protein content in each sample was detected by western blotting with anti-myc and anti-β-actin antibody.

By increasing the transfected plasmid by 2-fold and protein loading by 10-fold, the expression of zebrafish Ssat1b and Ssat1c in cells cultured in normal medium could be observed by western blotting. We used these conditions to observe protein stability inside cells. After adding cycloheximine to the culture medium, translation of Ssat1 was stopped and the protein was subsequently turned over. As shown in Figure 4A, the Ssat1b protein was quickly turned over after translation was stopped (Fig. 5A, lanes 3–5). Protein degradation was mediated by proteasome, since the addition of MG132 prevented degradation (Fig. 5A, lane 2). In addition, the presence of spermidine improved the stability of Ssat1b up to 6 hr (Fig. 5A, lanes 6–8). On the other hand, Ssat1a and Ssat1c were more stable and no obvious protein degradation occurred within 6 h (Fig. 5).

The 10 *ssat1a* and *ssat1b* chimeric genes were also applied to identify the critical regions responsible for rapid degradation (Fig. 2B). Our results indicated that none of the chimeric proteins, which contain the C-terminal regions of Ssat1a, turned over rapidly without spermidine treatment, such as Ssat1b467a, Ssat1b332a, and Ssat1aba shown here (Fig. 5B, lanes 1–3). Moreover, the chimeric proteins, which contain the C-terminal regions of Ssat1b, did not undergo rapid degradation without spermidine treatment, unless they contain more than 70 residues from the Ssat1b C-terminal region, such as Ssat1a332b (Fig. 5B, lanes 4–6) or Ssat1a248b (data not shown). The results, which are summarized in Table 1, indicated the last 70 residues of Ssat1b are important for the regulation of protein stability.

### The Enzyme Activities of Zebrafish *Ssat1*

The kinetic studies indicated all 3 Ssat1 isoenzymes were bioactive and could use both spermidine and spermine as substrates. However, the substrate preference of these isoenzymes was different. Ssat1b had similar K_m_ values for spermidine and spermine, while Ssat1a had a smaller K_m_ toward spermidine and Ssat1c had a smaller K_m_ for spermine (Table 2). Ssat1a and Ssatb had a better *k*_cat_/K_m_ value for spermidine than that for spermine, indicating these enzymes were more efficient in spermidine catabolism. In contrast, Ssat1c had a similar *k*_cat_/K_m_ ratio for spermidine and spermine.

**Table 2:** Enzyme kinetics of zebrafish family of Ssat1 proteins.

	Ssat1a		Ssat1b		Ssat1c	
	Km (µM)	Kcat/Km (M^−1^•sec^−1^)	Km (µM)	Kcat/Km (M^−1^•sec^−1^)	Km (µM)	Kcat/Km (M^−1^•sec^−1^)
Spermidine	182±20	1.222±0.433 × 10^5^	90±32	3.42±0.41 × 10^4^	232±42	1.5±0.5 × 10^3^
Spermine	55±15	6.24±0.35 × 10^4^	91±26	4.3±0.3 × 10^3^	139±37	1.9±0.6 × 10^3^

### Protein-protein Interactions of Zebrafish Family of *Ssat1* Proteins

**Figure 6:**
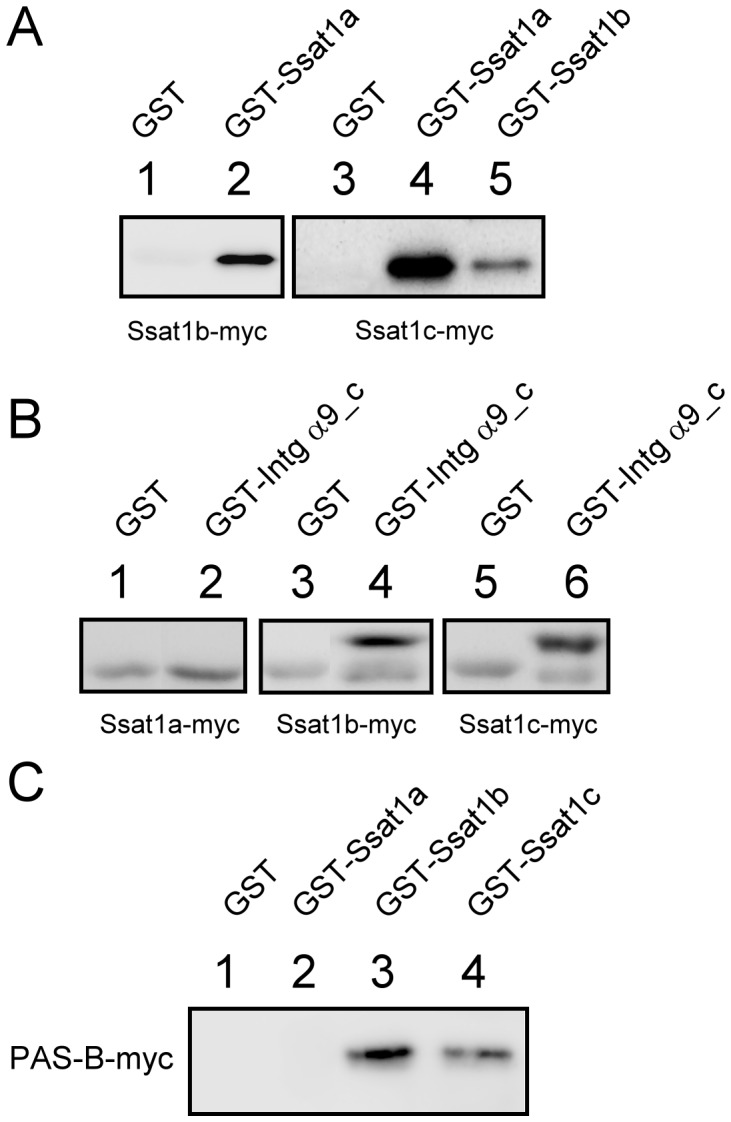
Protein-protein interactions of zebrafish family of Ssat1 proteins. (A) GST, GST-Ssat1a, GST-Ssat1b, and GST-Ssat1c were purified from bacteria. Lysates of cells transfected with the plasmid encoding Ssat1b were incubated with GST (Lane 1) or GST-Ssat1a (Lane 2). Lysates of cells transfected with the plasmid encoding Ssat1c were incubated with GST (Lane 3), GST-Ssat1a (Lane 4), or GST-Ssat1b (Lane 5). (B) GST and GST fused with the cytosolic domain of zebrafish Integrin 9α (GST-Intg9α_c) were purified from bacteria. Lysates of cells transfected with the plasmid encoding Ssat1a (lanes 1 and 2), Ssat1b (lanes 3 and 4), or Ssat1c (lanes 5 and 6) were incubated with GST (lanes 1, 3 and 5) or GST-Intg9α_c (lanes 2, 4 and 6). (C) Lysates of cells transfected with the plasmid encoding the myc-tagged PAS-B domain of Zebrafish Hif-1α was incubated with GST (lane 1), GST-Ssat1a (lane 2), GST-Ssat1b (lane 3), or GST-Ssat1c (lane 4). Bound proteins in each sample were pulled down with glutathione Sepharose 4B beads and analyzed by western blotting.

The structures of mammalian Ssat1s indicate the homodimer structure is essential for enzyme activity [33], [34]. Since Ssat1a, Ssat1b, and Ssat1c are co-expressed in several zebrafish organs and their primary sequences are largely identical, the formation of heterodimers is possible. Here, GST pull-down experiments were applied to test this hypothesis. The GST-Ssat1a fusion protein was able to pull down Ssat1b-myc and Ssat1c-myc while GST itself could not (Fig. 6A). In addition, GST-Ssat1b interacted with Ssat1c-myc, suggesting the 3 zebrafish family of Ssat1 proteins could assemble into homodimers or heterodimers.

It has been reported that human SSAT1 interacts with the cytosolic domain of integrin α9 to enhance cell migration [15]. A recombinant GST fusion to the cytosolic domain of zebrafish integrin α9 (GST-Intg α9) was used to study interactions with zebrafish family of Ssat1 proteins. Ssat1b and Ssat1c interacted with integrin α9, but Ssat1a did not (Fig. 6B).

Human SSAT1 also binds to the PAS-B (Per-ARNT-Sim) domain of HIF-1α, facilitating its degradation [16]. A DNA fragment encoding zebrafish Hif-1α PAS-B was cloned into pcDNA3.1/myc-His and transfected into *HEK293T* cells. Cell lysates were extracted and incubated with GST-fused Ssat1 proteins. The results of GST pull-down experiments indicated that Ssat1b and Ssat1c interacted with the Hif-1α PAS-B domain but Ssat1a did not (Fig. 6C).

## Discussion

Despite reports of several polyamine acetyltransferases in microbes, their sequences were neither similar to each other nor to animal *ssat-like* genes [35], [36], [37], [38], [39], suggesting different evolutionary origins. In this work, 29 *ssat* homologs from 10 deuterostomia species were identified and analyzed. Comparing with *ssat2* genes and invertebrates’ *ssat-like* genes, *ssat1* genes are present only in vertebrates and highly conserved to each other (Fig. 1). Previous studies indicated that human SSAT2 and Ssat-like enzymes from several organisms do not involve polyamine catabolism, because these enzymes prefer to use thialysine as their substrate [40], [41], [42]. Therefore, the polyamine interconversion pathway might only be evolved in the vertebrate lineage.

In humans, *SSAT1* mRNA is transcribed in almost every tissue with 2 splicing forms [20]. In addition to the normal mRNA, one transcriptional variant (*SSAT-X* mRNA), which cannot produce bioactive SSAT1 due to its incorporation of an extra exon from intron 3, is accumulated in hypoxic or iron-deficient cells [20]. When cellular polyamine level increasing, the transcription of *SSAT1* is induced [17], [18] while the alternative spliced *SSAT-X* mRNA is reduced [19]. In zebrafish, none of these *ssat1* genes was induced by polyamine analog (Fig. 3A) and RT-PCR found no sign of alternative transcript splicing (Fig. 3). Zebrafish thus does not appear to regulate Ssat1 activity through mRNA transcription and alternative splicing.

A previous report indicated that an RNA binding protein, which might bind to the stem-loop structures in the 5′ and 3′ regions of the human *SSAT1* ORF, strictly inhibits *SSAT1* mRNA translation [22]. A nucleolin isoform was recently identified as a key factor to stabilize the 5′ stem-loop structure of *SSAT1* ORF. Addition of polyamine enhances the turnover of nucleolin thus significantly restore translation [43]. In zebrafish, the stringent inhibition of background translation was only observed in *ssat1b* and *ssat1c* (Fig. 4A). By assaying a series of chimeric zebrafish *ssat1* genes, we found that the both 5′ and 3′ regions of *ssat1b* were important for such regulation (Fig. 4B) that seems to be in accordance with the previous study [22]. However, it is difficult to explain that altering the 5′ or 3′ mRNA structures by replacing the first 332 or the last 124 nucleotides of *ssat1b* with that of *ssat1a* did not affect the regulatory pattern of *ssat1b* (Fig. 4B, a332b and b389a). Moreover, attempts to search similar stem-loop structures of human *SSAT1* in zebrafish *ssat1b* and *ssat1c* were also failed.

A recent study of yeast antizyme (Az) might provide another explanation. Like Ssat1, Az is also a homeostatic feedback regulator of polyamines, since the activity of ornithine decarboxylase, a key enzyme in polyamine biosynthesis pathway, is readily down-regulated in the presence of Az [2]. It is interesting to note that the mRNA translation of *Az* is regulated in response to the cellular polyamine level, too. A unique polyamine-dependent +1 ribosomal frameshifting regulatory mechanism involving the 5′ region of *Az* mRNA is conserved in many *Az* orthologues [44]. In addition, Kurian et al. recently found another regulatory mechanism of Az synthesis involving the 3′ region of its mRNA. In fact, it is the encoded amino acid sequence responsible for such kind of regulation, since the nascent Az will act as a sensor of polyamine and regulate the completion of its own translation [45]. Until now, it has been unclear whether nascent Ssat1 serves as a polyamine sensor and regulates its own translation. However, it was known that the amino acid residues in the C-terminus of human SSAT1 are also important for translational regulation [23]. Deleting more than 5 residues from the C-terminus has a significant impact, but silent mutations, in which several nucleotides in the 3′ end were modified without changing the coding sequence, retain polyamine responsiveness [22]. Nevertheless, further study is needed to test this hypothesis.

The fourth regulatory mechanism of human SSAT1 is modulation of protein stability. Binding of polyamines or polyamine analogs changes the configuration of the SSAT1 protein. Therefore, SSAT1 is prevented from ubiquitination and becomes stable [23], [25]. Extensive effort has led to the identification of key residues responsible for this regulation [24]. The MATEE sequence in the C-terminus is important. SSAT1 stabilizes in the absence of polyamine when M^167^, E^170^, and E^171^ are substituted or the last 2 residues (E^170^ and E^171^) are deleted. SSAT1, however, carries on rapid degradation even in the presence of polyamine when point mutations are made at R^7^, C^14^, R^19^, H^126^, K^141^, E^152^, or R^155^. These residues in human SSAT1 are largely conserved in zebrafish Ssat1b, except the MATEE sequence is replaced by MESEE in the C-terminus (Fig. 2A). Note that M^167^, E^170^, and E^171^ are conserved in zebrafish Ssat1a and Ssat1b, but Ssat1a does not turn over rapidly (Fig. 5A). The chimeric enzyme Ssat1a453b, which preserved the last 20 residues of Ssat1b, was as stable as Ssat1a, suggesting more residues may be involved in this mechanism. The results of Ssat1a332b indicated that the last 70 residues of Ssat1b were important for its rapid degradation (Fig. 5B). Only 14 residues differ in the last 70 amino acids of Ssat1a and Ssat1b. Further study is needed to identify the residues responsible for regulating protein stability.

All zebrafish family of Ssat1 proteins were active enzymes, though their substrate preferences and catalytic efficiencies differed. Moreover, our data also indicated these zebrafish Ssat1 proteins form heterodimers (Fig. 6A). Their kinetics may be more complex due to heterodimerization. Considering the differences of these Ssat1 proteins in their expression profile, regulation patterns and enzyme activities, zebrafish might be able to fine-tune the metabolism of polyamine to fit the physiological requirements in different organs by expressing different Ssat1 proteins.

Integrins are cell surface proteins that mediate cell-cell communication and cell morphology. Integrin α9, a mammalian specific form [46], is stimulated by extracellular signals, such as tenascin C [47], osteopontin [48], and vascular cell adhesion molecules-1 [49], and involved in embryogenesis [50], lymphangiogenesis, and wound healing [51]. It has been reported that overexpression of human SSAT1 enhances cell migration mediated by integrin α9 [15]. The first 20 amino acids of SSAT1 is crucial, since they could bind to the cytosolic domain of integrin α9 thus regulates the migration signaling [52]. In this study, we identified an integrin α9 orthologue in zebrafish. Although the length of the extracellular region differs significantly between human and zebrafish integrin α9, the sequences of their cytosolic domains are largely identical (Fig. S4). By using GST-pull down experiments, we confirmed that zebrafish integrin α9 interacts with Ssat1b and Ssat1c, but not Ssat1a (Fig. 6B). It is worth noting that Ser^15^ of Ssat1b, Ssat1c, and human SSAT1 was replaced by Pro^15^ in Ssat1a. The structures of SSAT1 reveal a conserved α-helix located between residues 12 and 28 (Fig. 2A) [33]. Pro^15^ in Ssat1a may break the helix structure and thus interfere with the interaction between Ssat1a and integrin α9.

Hif-1α, a key regulator of oxygen homeostasis in all metazoans, is mainly regulated by an oxygen-sensing prolyl hydroxylase, which facilitates its rapidly degradation in proteosome [53]. A previous study has shown another oxygen- independent Hif-1α regulation mechanism that is triggered by the binding of human SSAT1 with the PAS-B domain of HIF-1α [16]. PAS domains, found in many proteins in all kingdoms of life, are structurally conserved protein-protein interaction modules [54]. Although the amino acid sequences in PAS-B domains of human and zebrafish Hif-1α are highly conserved (Fig. S5), it is interesting to note that only Ssat1b and Ssat1c, but not Ssat1a, were able to interact with the PAS-B domain of zebrafish Hif-1α (Fig. 6C). The sequence variants between these homologues may provide clues to identify the critical regions responsible for Hif-1α binding in the future.

Gene duplication is considered to be the major force of evolution [55], because new copies may acquire new functions by mutation (known as neofunctionalization) [56]. However, the fates of redundant genes might also include becoming pseudogenes (nonfunctionalization) or being preserved in a complementary partitioning of subfunctions (subfunctionalization) [56]. It is generally believed that 2 rounds of whole-genome duplication occurred during the intergradation of vertebrates from their deuterostome ancestors [57]. Interestingly, we noticed that not only *ssat1* but also *hif-1α* [53] and *integrin α9* [46] were evolved simultaneously in the vertebrate lineage. They might experience neofunctionalization to meet the physiological requirements of vertebrates.

In comparison with mammals, the ray-finned fishes underwent an extra round of whole-genome duplication, which caused the teleost radiation [58]. It seems that nonfunctionalization is the fate of the majority of duplicated *ssat1* genes, as is the case in medaka, stickleback, takifugu, and tetraodon. Thus there is only one *ssat1* left in these species. However, zebrafish, which contains not one but three *ssat1* homologues, is an exception. The phylogenetic analysis indicates that all zebrafish *ssat1* homologues are derived from a common ancestor (Fig. 1). The remnants of vertebrates’ *ssat1* syntenic genes are found scattering in the loci of each homologue. For example, *acot9* and *apoo* are clustered with *ssat1a* in chromosome 24, and *prdx4* is closely located near *ssat1b* and *ssat1c* in chromosome 5 (Fig. S1). It is worth to note that the sequences between *ssat1b* and *ssat1c* are more similar to each other (Fig. S2). Further, Ssat1b and Ssat1c also have similar translational regulation pattern and protein-protein interaction relationships with Hif-1α and Integrin α9. These observations suggest that *ssat1a* might be one of the products from the teleosts’ whole genome duplication while the other one underwent a local duplication to form *ssat1b* and *ssat1c* later on.

Our results suggest that zebrafish *ssat1* homologues are paralogous genes which experienced subfunctionalization in their function and regulation. It is worth noting that only Ssat1b and Ssat1c, but not the polyamine-insensitive Ssat1a, are able to interact with integrin α9 and Hif-1α. Thus these signal pathways could be regulated by Ssat1 in response to cellular polyamine level. Besides polyamine catabolism, it might be the key feature that allows Ssat1 to coordinate certain physiological responses in vertebrates, such as fine-tuning the advanced immune system and the homeostasis of polyamine and hypoxia.

By characterizing properties of zebrafish family of Ssat1 proteins and the artificial chimeric enzymes, our data revealed some correlations between their sequences and functions that may provide valuable information for studies of the translational regulatory mechanism, protein stability, and physiological functions of Ssat1 in the future.

## Supporting Information

Figure S1 **Chromosomal localizations of human** ***SSAT1*** **and zebrafish** ***ssat1*** **homologues.**
(TIF)

Figure S2 **Alignment of zebrafish** ***ssat1*** **homologues cDNA sequence.** The cDNA sequences were aligned by MegAlign (Lasergene) with the ClustalW method. The conserved residues are shaded black. The denoted amino acid sequences underneath cDNA sequences are consensus residues in all three homologues, while the encoded amino acids which are not conserved in all three homologues are denoted by dash symbols.
(TIF)

Figure S3 **Translational regulation of** ***ssat1*** **genes in zebrafish cells.** *ZF4* cells were transiently transfected with the plasmid encoding myc-tagged full-length human SSAT1, zebrafish Ssat1a, or Ssat1b. After incubation for 12 h, transfected cells were treated with 10 µM DENSPM, 20 µM MG132, or vehicle for 24 h. Cell lysates (5 µg total protein in each lane) were prepared and the Ssat1 protein content in each sample was detected by western blotting with anti-myc antibody.
(TIF)

Figure S4 **Sequence alignment of integrin α9.** The amino acid sequences of human (NP_002198), mouse (NP_598482), and zebrafish integrin α9 (XP_003199805) were aligned by MegAlign (Lasergene) with the ClustalW method. The conserved residues are shaded in black. The cytosolic domains are marked with a red box.
(TIF)

Figure S5 **Sequence alignment of human, mouse and zebrafish Hif-1α.** The amino acid sequences of human (NP_001521), mouse (NP_034561), and zebrafish Hif-1α (AAQ91619) were aligned by MegAlign (Lasergene) with the ClustalW method. The conserved residues are shaded in black. The PAS-B domain is underlined in red.
(TIF)

Table S1 **Oligonucleotide primers used in this work.**
(DOC)
