# Supplementary figures and images for: Duplication and Diversification of the Spermidine/Spermine N1-acetyltransferase 1 Genes in Zebrafish
Source: PLoS One. 2013 Jan 11;8(1):e54017. doi: 10.1371/journal.pone.0054017 (PMC3543422; doi:10.1371/journal.pone.0054017)

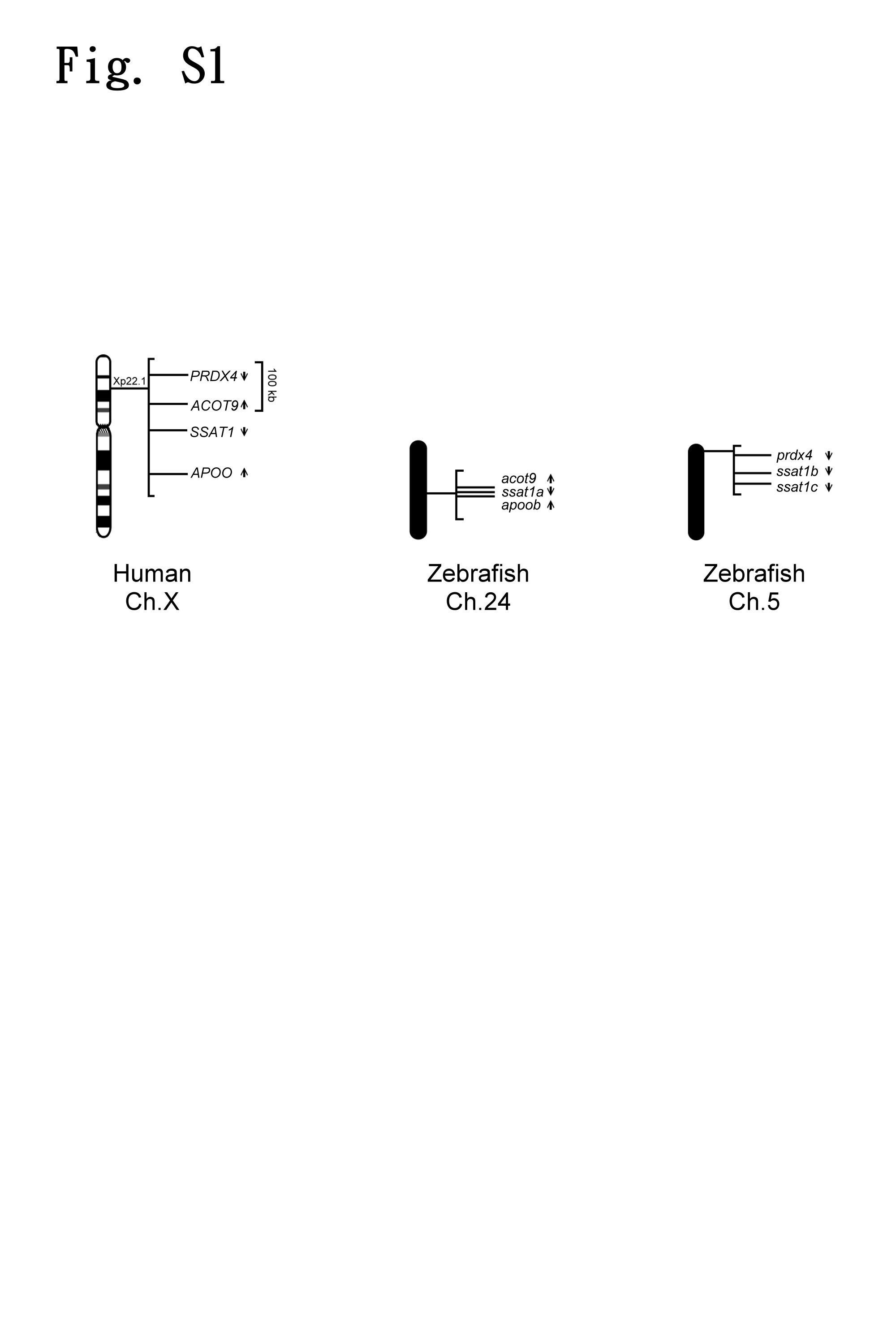

Supplement: Figure S1 — Chromosomal localizations of human SSAT1 and zebrafish ssat1 homologues. (TIF) [file pone.0054017.s001.tif]

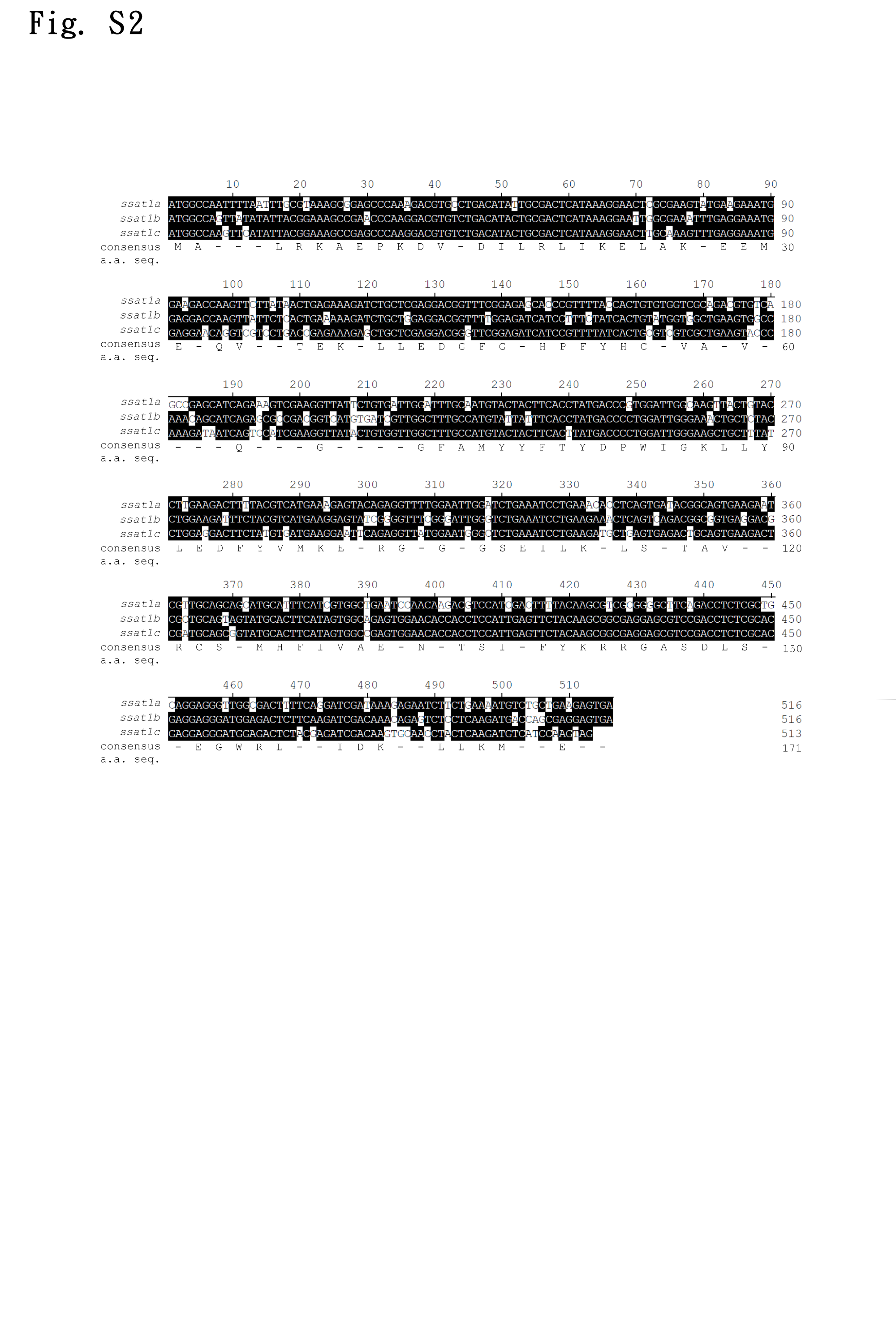

Supplement: Figure S2 — Alignment of zebrafish ssat1 homologues cDNA sequence. The cDNA sequences were aligned by MegAlign (Lasergene) with the ClustalW method. The conserved residues are shaded black. The denoted amino acid sequences underneath cDNA sequences are consensus residues in all three homologues, while the encoded amino acids which are not conserved in all three homologues are denoted by dash symbols. (TIF) [file pone.0054017.s002.tif]

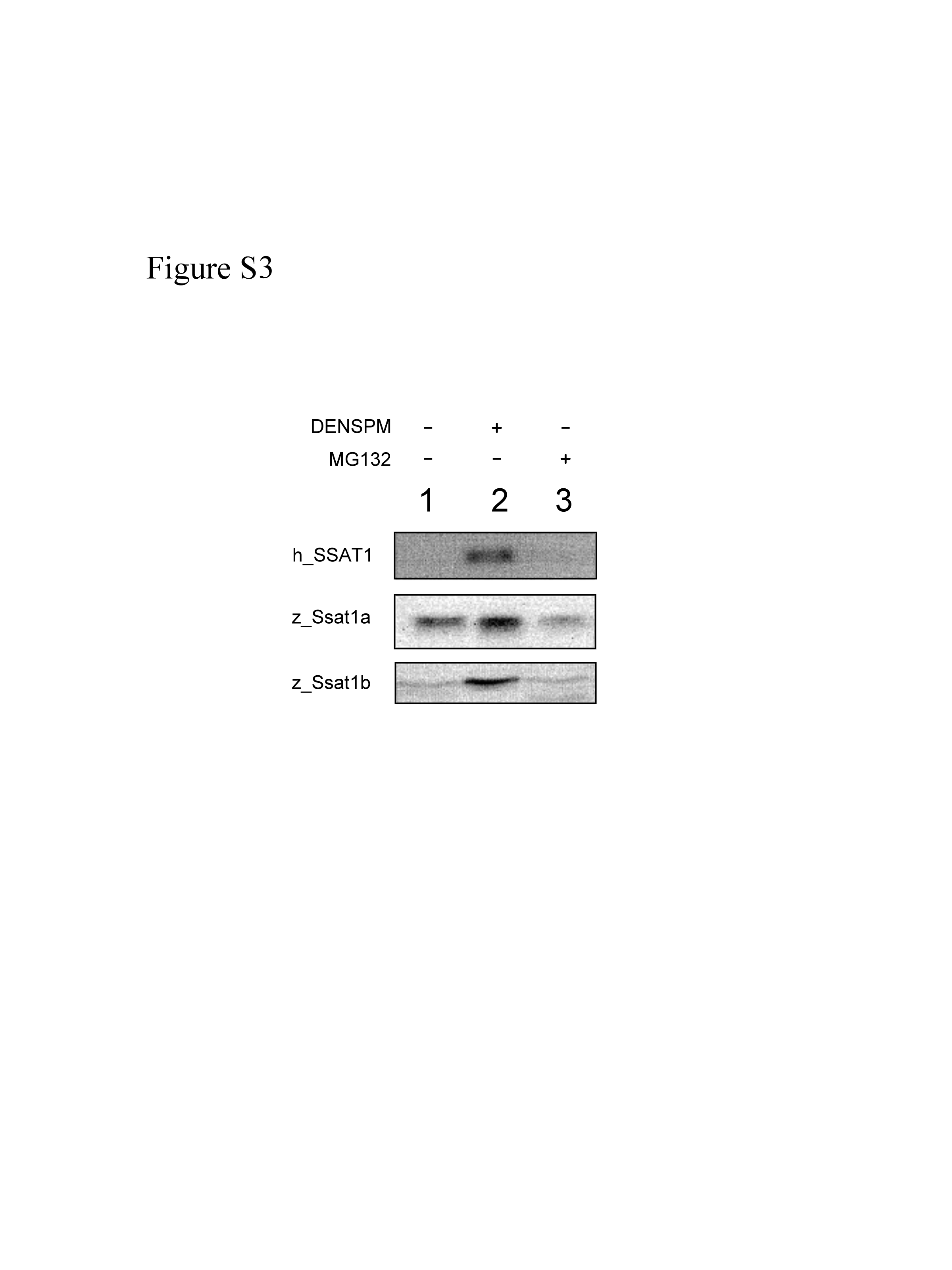

Supplement: Figure S3 — Translational regulation of ssat1 genes in zebrafish cells. ZF4 cells were transiently transfected with the plasmid encoding myc-tagged full-length human SSAT1, zebrafish Ssat1a, or Ssat1b. After incubation for 12 h, transfected cells were treated with 10 µM DENSPM, 20 µM MG132, or vehicle for 24 h. Cell lysates (5 µg total protein in each lane) were prepared and the Ssat1 protein content in each sample was detected by western blotting with anti-myc antibody. (TIF) [file pone.0054017.s003.tif]

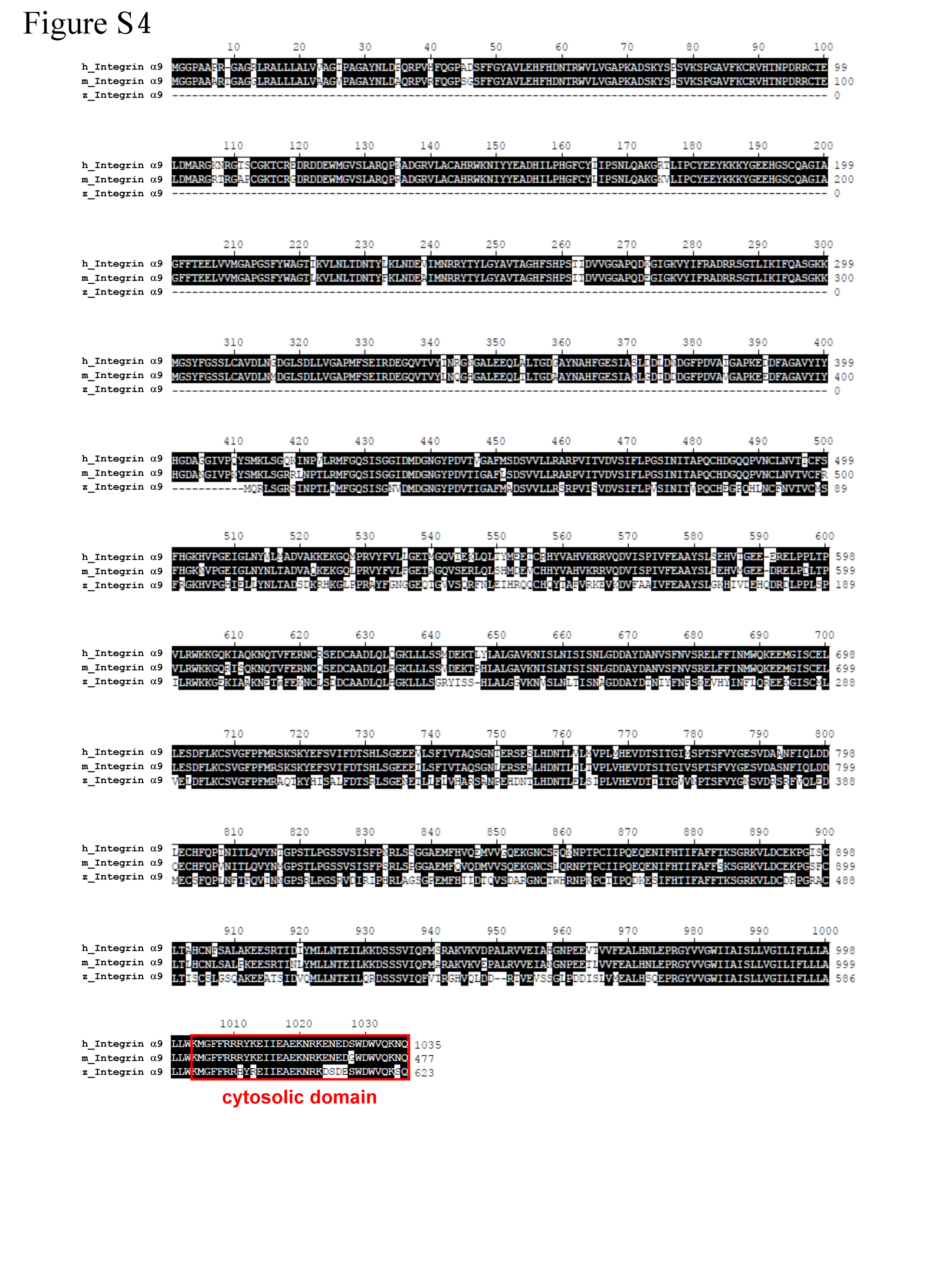

Supplement: Figure S4 — Sequence alignment of integrin α9. The amino acid sequences of human (NP_002198), mouse (NP_598482), and zebrafish integrin α9 (XP_003199805) were aligned by MegAlign (Lasergene) with the ClustalW method. The conserved residues are shaded in black. The cytosolic domains are marked with a red box. (TIF) [file pone.0054017.s004.tif]

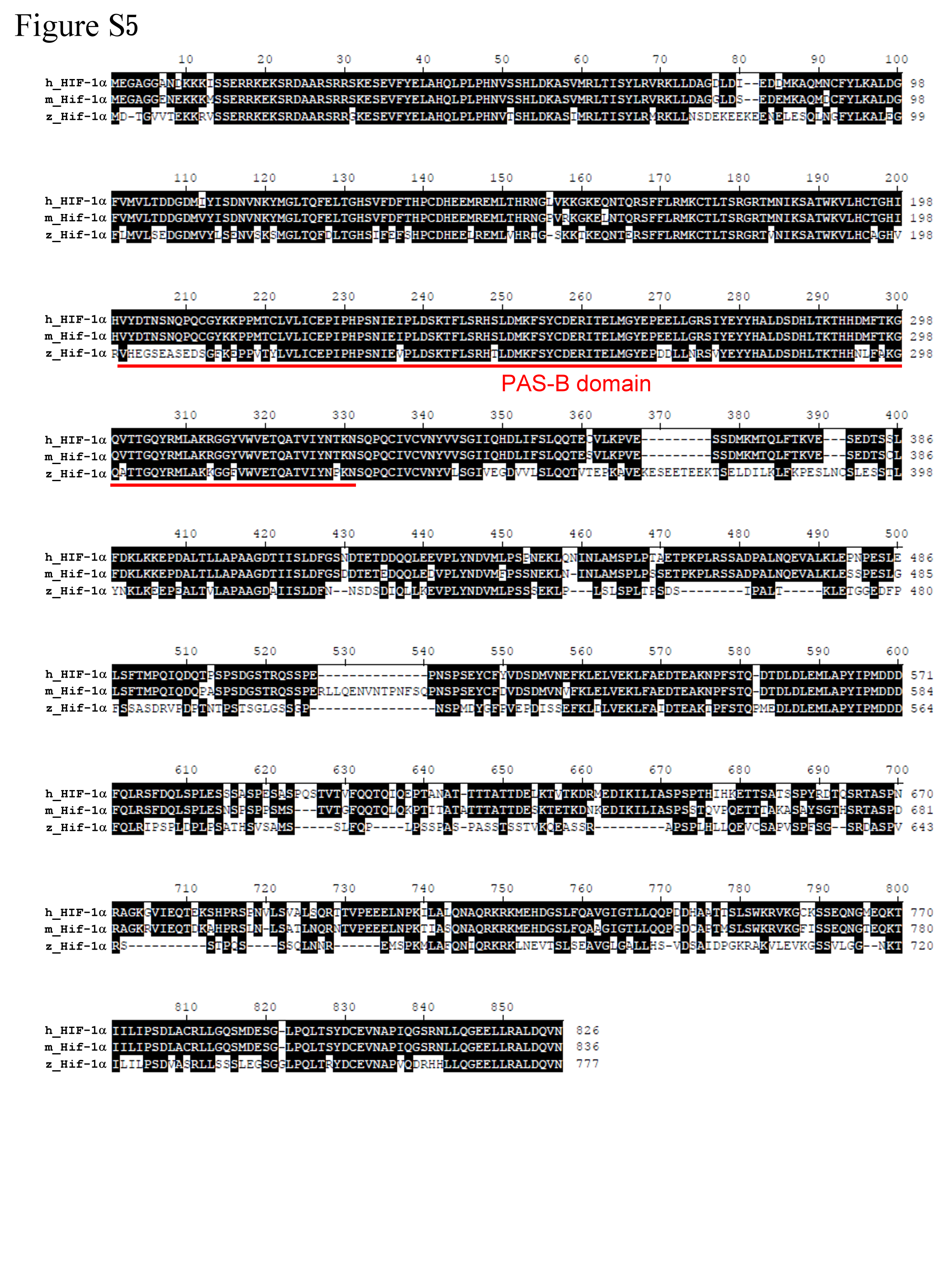

Supplement: Figure S5 — Sequence alignment of human, mouse and zebrafish Hif-1α. The amino acid sequences of human (NP_001521), mouse (NP_034561), and zebrafish Hif-1α (AAQ91619) were aligned by MegAlign (Lasergene) with the ClustalW method. The conserved residues are shaded in black. The PAS-B domain is underlined in red. (TIF) [file pone.0054017.s005.tif]
