# Supplementary material for: Duplication and Diversification of the Spermidine/Spermine N1-acetyltransferase 1 Genes in Zebrafish
Source: PLoS One. 2013 Jan 11;8(1):e54017. doi: 10.1371/journal.pone.0054017 (PMC3543422; doi:10.1371/journal.pone.0054017)
Supplement: Table S1 — Oligonucleotide primers used in this work. (DOC) [file pone.0054017.s006.doc]

**Table S1: Oligonucleotide primers used in this work**

| Entry | target gene | sequence | direction |
| --- | --- | --- | --- |
| 1 | *β-actin* | CGGCTCCGGTATGTGC | forward |
| 2 | *β-actin* | TCTGTTGGCTTTGGGATTC | reverse |
| 3 | *ssat1a* | GAATTCATGGCCAATTTTAATTTG | forward |
| 4 | *ssat1a* | CTCGAGCTCTTCAGCAGACATTT | reverse |
| 5 | *ssat1b* | GAATTCATGGCCAGTTATATATTAT | forward |
| 6 | *ssat1b* | CTCGAGCTCCTCGCTGGTCATCTT | reverse |
| 7 | *ssat1c* | GAATTCATGGCCAAGTTCAT ATTACGG | forward |
| 8 | *ssat1c* | CTCGAGCTTGGATG ACATCTTGAGTAGG | reverse |
| 9 | *h_SSAT1* | GAATTCATGGCTAAATTCGTGATC | forward |
| 10 | *h_SSAT1* | CTCGAGCTCCTCTGTTGCATTTT | reverse |
| 11 | *a248b/b248a* | GGGTCATAGGTGAA | reverse |
| 12 | *a332b/b332a* | TCTGAAATCCTGAA | forward |
| 13 | *a374b/b389a* | TGCACTTCATAGTGGC | forward |
| 14 | *a453b/b467a* | AGGAGGGATGGAGACT | forward |
| 15 | *Integrin α9* | GAATTCAAGATGGGATTCTTCA | forward |
| 16 | *Integrin α9* | CTCGAGTTGGCTCTTCTGGACC | reverse |
| 17 | *hif-1α* | GAATTCGTGCACGAGGGCAGCGA | forward |
| 18 | *hif-1α* | CTCGAGATTCTTGGGGTTGTAGATTAC | reverse |
